# Integrative pan-cancer analysis of MEK1 aberrations and the potential clinical implications

**DOI:** 10.1038/s41598-021-97840-0

**Published:** 2021-09-15

**Authors:** Zhiyang Zhou, Bi Peng, Juanni Li, Kewa Gao, Yuan Cai, Zhijie Xu, Yuanliang Yan

**Affiliations:** 1 Department of Breast Surgery, Xiangya Hospital, Central South University, Changsha, Hunan China; 2 Department of Pathology, Xiangya Hospital, Central South University, Changsha, 410008 Hunan China; 3 Department of Pharmacy, Xiangya Hospital, Central South University, Changsha, 410008 Hunan China; 4 National Clinical Research Center for Geriatric Disorders, Xiangya Hospital, Central South University, Changsha, 410008 Hunan China

**Keywords:** Cancer, Data processing

## Abstract

Alterations of mitogen-activated protein kinase kinase 1 (MEK1) are commonly associated with tumorigenesis, and MEK1 is thought to be a suitable targeted therapy for various cancers. However, abnormal MEK1 alterations and their relevant clinical implications are unknown. Our research comprehensively analyzed the MEK1 alteration spectrum and provided novel insight for targeted therapies. There were 7694 samples covering 32 types of cancer from The Cancer Genome Atlas (TCGA) database. They were used to conduct an integrative analysis of MEK1 expression, alterations, functional impacts and clinical significance. There was a dramatic difference in the alteration frequency and distribution and clinical implications in 32 types of cancer from the TCGA. Skin cutaneous melanoma (SKCM) has the most alterations and has therapeutic targets located in the protein kinase domain, and the growing expression of SKCM is positively related to patient prognosis. MEK1 expression in lung adenocarcinoma (LUAD), kidney renal papillary cell carcinoma (KIRP), esophageal carcinoma (ESCA) and liver hepatocellular carcinoma (LIHC) is decreased, which is associated with better prognosis, while MEK1 expression in thymoma (THYM), stomach adenocarcinoma (STAD), kidney renal clear cell carcinoma (KIRC), testicular germ cell tumors (TGCTs) and head and neck squamous cell carcinoma (HNSC) is increased, which is associated with better prognosis. Mesothelioma (MESO) has the second highest alterations but has no therapy targets. This study provided a great and detailed interpretation of MEK1 expression, alterations and clinical implications in 32 types of cancer and reminded us to fill the gap in MEK1 research from a new perspective.

## Introduction

Mitogen-activated protein kinase kinase 1 (MEK1), a small molecular substance belonging to the family of receptor tyrosine kinases (RTKs), has a key function in the mitogen-activated protein kinase (MAPK) cascade^1^, which primarily contains the RAS/MAPK signaling pathway, JNK signaling pathway, p38MAPK signaling pathways and extracellular signal regulated kinase (ERK) signaling pathways^2–4^. Human MEK1 is composed of 393 amino acids, which include a trifunctional N-terminal sequence, a kinase catalytic domain and a C-terminal sequence^5^. MEK1 is one of the downstream effectors of RAS/MAPK signaling pathways, which not only governs normal cell proliferation, survival, and differentiation, but also triggers excessive cell division and promotes the occurrence and development of tumors^6^. Moreover, MEK1 also serves as a key signal node to regulate isoforms ERK1 and ERK2 and deliver signals to the nucleus^7^. In this signaling pathway, the adaptor proteins and guanine nucleotide exchange factors, such as Son of Sevenless (SOS), are recruited to promote the combination of RAS and GTP. After then, RAS-GTP catalyzes the activation of RAF kinases, including ARAF, BRAF and CRAF. Activated RAF kinases promote MEK phosphorylation and activation. ERK1/2 are phosphorylated and activated by phosphorylated MEK1/2, and then promote the expressions of transcription factors to collectively regulate multiple cellular and biological functions during cancer development and development^8^.

Because of the aforementioned MEK1 regulating mechanism, MEK1 is able to be regarded as an effective therapeutic target and plays a vital role in oncogenesis processes^9^. MEK1 can be considered to be an oncogene, resulting in a series of tumorigenesis and alloplasia. It was previously found that a diverse set of MEK1 gene mutations are associated with various somatic tumors such as melanoma, histiocytic neoplasms, colorectal cancer and lung cancer^10^.

Accordingly, MEK1 inhibitors have been deemed to be an attractive strategy for the treatment of numerous cancers due to their crucial functions. Many MEK1 inhibitors have been naturally created and extensively used to treat a wide range of cancers due to their preclinical and clinical potentialities^11^. Méndez-Martínez et al. found that MEK1 inhibitors for the treatment of metastatic BRAF-mutant cutaneous melanoma and NRAS mutant melanoma were ground-breaking therapeutic regimens, respectively^12^. Kim et al. found that MEK1 inhibitors contributed to the treatment of NSCLC and confirmed their anticancer activity^13^. Additionally, MEK1 inhibitors also had a positive influence in advanced thyroid cancer (THCA), neurofibromatosis type 1 (NF1), BRAF-mutant pediatric low-grade gliomas, colon cancer and histiocytic neoplasms^10^. Thus, many tumors are related to MEK1, which should receive more attention and be explored deeper. Surprisingly, there are a lot of MEK1 inhibitors that have been approved for the treatment of various cancers by the Food and Drug Administration (FDA). For instance, the combination of highly selective inhibitors of MEK1/2, including trametinib, cobimetinib and binimetinib, and BRAF inhibitors have been served as the FDA-approved strategies for the treatment of BRAF V600E-mutant melanoma^14^. On April 10, 2020, selumetinib received FDA approval for pediatric patients with neurofibromatosis type 1, and they must be at least 2 years old, symptomatic and unable to surgery^15^. Moreover, Researches on other MEK1 inhibitors are also performing, such as PD-0325901, TAK-733 and Refametinib^11^.

However, prior studies have failed to identify the comprehensive and acknowledged profiling and significance of MEK1 due to a limited number of samples and/or limited types of cancer. We conducted this research using bioinformatics tools such as The TCGA and Kaplan–Meier analysis. Systematic mutation, copy number variants (CNVs) and expression were obtained across 32 different types of cancer from the TCGA. Then, we take advantage of Kaplan–Meier curves to analyze the clinical significance and promising insights according to the corresponding alterations.

## Materials and methods

### Data acquisition from various bioinformation databases

MEK1 expression in 53 normal tissues was acquired using genotype-tissue expression (GTEx), whose primary functions include the collection of the genetic impact on transcriptome and gene regulation mechanism, and MEK1 expression data were log10 transformed^16^. Then, cBioPortal was applied to obtain MEK1 mRNA expression data. cBioPortal is very useful for the exploration, visualization, and analysis of cancer genomics and clinical data^17^. We further utilized GEPIA, which is a practical and comprehensive website contributing to gene expression profiling and interactive analysis, to compare the transcriptional levels between 32 types of cancer and matched normal tissues^18^. Moreover, MEK1 protein expression was extracted from the first reverse phase protein array (RPPA) dataset containing more than 8000 samples of 32 types of cancer from TCGA^19^.

To determine MEK1 in terms of mutational and clinical significance, we utilized cBioPortral again to obtain these related data. The mutation data included indels, single-nucleotide variants (SNVs) and CNVs that contained amplification and deep deletion. The log ratio value of CNVs was set as follows: − 2/ − 1 = deletion, 0 = diploid, 1 = gain, and 2 = amplification. Then, the exploration of the clinical significance was mainly based on Kaplan–Meier curves. Kaplan–Meier curves are a method to predict the future and a convenient way to evaluate the effect of genes on patient survival^20,21^. Survival indexes such as overall survival (OS) and progression-free survival (PFS) were primarily used to analyze the association with MEK1 alterations. The 95% confidence intervals and the hazard ratios were plotted as forest plots.

### Data analysis

IBM SPSS Statistics v26.0.0.5 (https://www.ibm.com/cn-zh/products/spss-statistics) was used for data analysis. Student’s t-tests, Cox regression analysis and linear regressions were appropriately applied. A p value less than 0.05 was considered significant. All cancer samples were statistically analyzed. A single sample at the genetic level was classified into two major categories: altered and unaltered. The Onco Query Language (OQL) was mainly applied.

## Results

### MEK1 expression across 32 types of cancer

**Figure 1:**
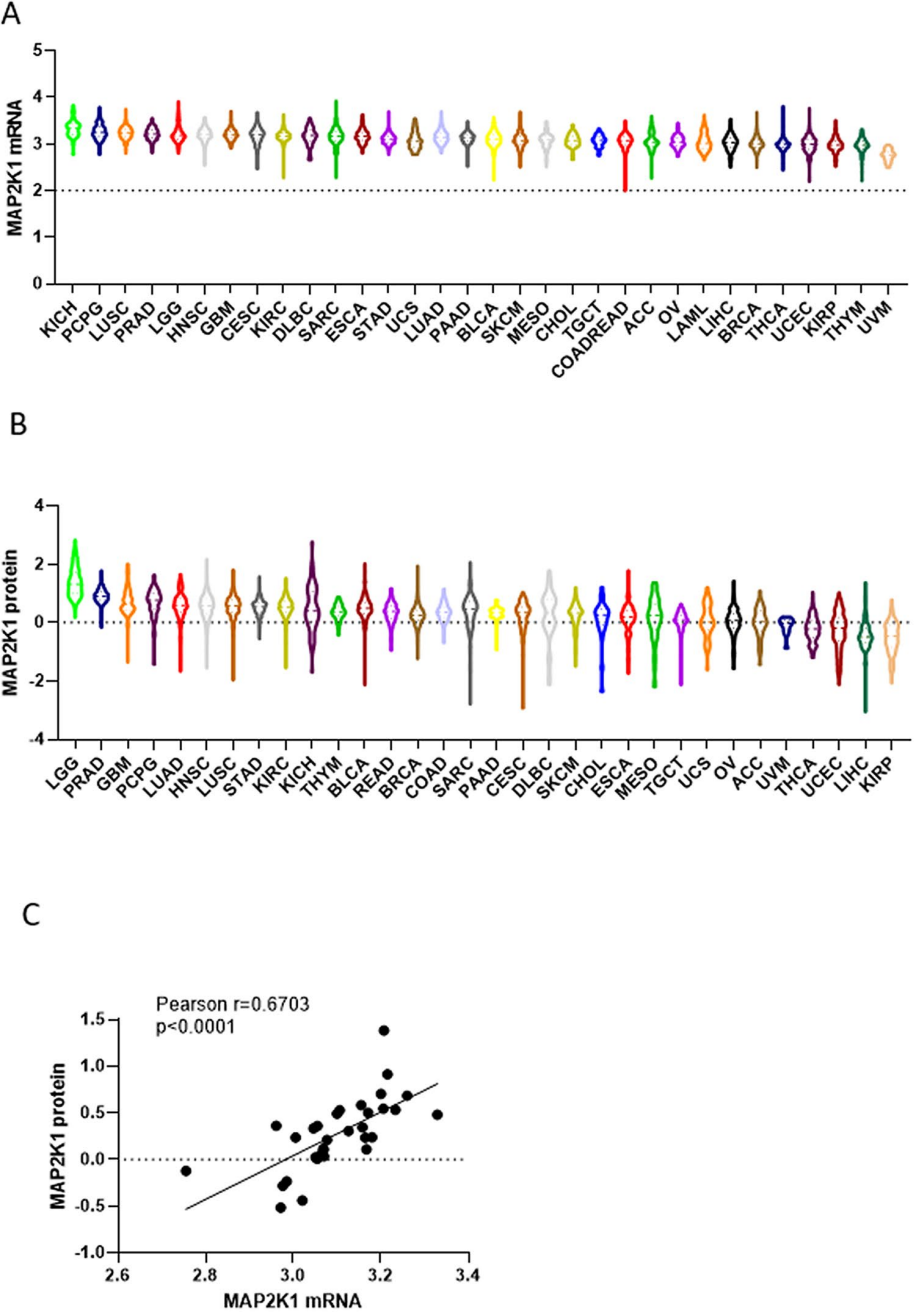
The expression of MEK1 mRNA and protein from The Cancer Genome Atlas (TCGA). (**A**) MEK1 mRNA expression in pancancer. (**B**) MEK1 protein expression in pancancer. (**C**) MEK1 mRNA expression had a positive correlation with MEK1 protein expression across 32 types of cancer types.

Prior studies have substantiated that RAS/MAPK pathway overactivation promotes overexpression, leading to changes in MEK1 expression in various tumors^17^, but these results were far from sufficient. Studies on MEK1 expression remain a daunting challenge. Thus, we utilized GTEx to obtain an integrative analysis of MEK1 expression in pancancer and 53 normal tissues, and then we compared abnormal MEK1 expression with normal. Supplementary Figure S1A shows that MEK1 was differentially and markedly expressed in different normal tissues. The top three expression levels were as follows: cell EBV-transformed lymphocytes, brain-cerebellar hemisphere and brain-frontal cortex (BA9), respectively. Conversely, MEK1 was expressed at low levels in the kidney cortex, left ventricle of the heart and pancreas. Then, the MEK1 mRNA expression levels were profiled in pancancer. The spectrum of MEK1 mRNA expression was dramatically variable, indicating that MEK1 harbored some distinct traits to be expressed widespread, such as diffuse large B cell lymphoma (DLBC), acute myeloid leukemia (LAML) and uterine carcinosarcoma (UCS); or to be expressed narrowly, such as KIRC, THCA and pancreatic carcinoma (PAAD). The generation of this difference may be associated with multiple subtypes in one cancer and diverse gene phenotypes (Fig. 1A). To determine the actual changes in MEK1 expression in pancancer, we made a comparison between tumors and matched normal tissues. Obviously, these expression changes were markedly differential. As shown in the Supplementary Figure S1B, 18 types of tumors were upregulated [bladder urothelial carcinoma (BLCA), cervical and endocervical cancers (CESC), cholangiocarcinoma (CHOL), colon adenocarcinoma (COAD), DLBC, HNSC, kidney chromophobe (KICH), lung squamous cell carcinoma (LUSC), PAAD, rectum adenocarcinoma (READ), sarcoma (SARC), skin cutaneous melanoma (SKCM), STAD, TGCT, THCA, KIRC, and THYM] , 12 types of tumors were downregulated [adrenocortical carcinoma (ACC), breast invasive carcinoma (BRCA), ESCA, glioblastoma multiforme (GBM), kidney renal papillary cell carcinoma (KIRP), LAML, brain Lower Grade Glioma (LGG), LIHC, LUAD, ovarian serous cystadenocarcinoma (OV), and uterine corpus endometrial carcinoma (UCEC)] and the results of remaining 3 types of cancer were invalid because of the limited number of samples [pheochromocytoma and paraganglioma (PCPG), uveal melanoma (UVM), and MESO]. Moreover, obviously increased expression of MEK1 was detected in DLBC, HNSC, SKCM, STAD and TGCT. In contrast, distinctly decreased expression of MEK1 was found in LAML and LIHC.

Because of the correlation between mRNA expression and protein expression^22^, we further analyzed the MEK1 protein expression in pancancer and these data obtained from the Cancer Proteome Atlas (TCPA)^19^. The results presented a striking similarity to MEK1 mRNA. Likewise, the profile of MEK1 protein expression was wide, and there were significant differences in different tumors (Fig. 1B). Furthermore, we observed that there was a high positive correlation between MEK1 mRNA and protein expression in pancancer (r = 0.6703, p < 0.0001). These observations implied that MEK1 gene expression was the key to driving and regulating protein expression and had a tremendous impact on tumor functions (Fig. 1C).

### The specific mutation frequency and distribution of MEK1 in pancancer

**Figure 2:**
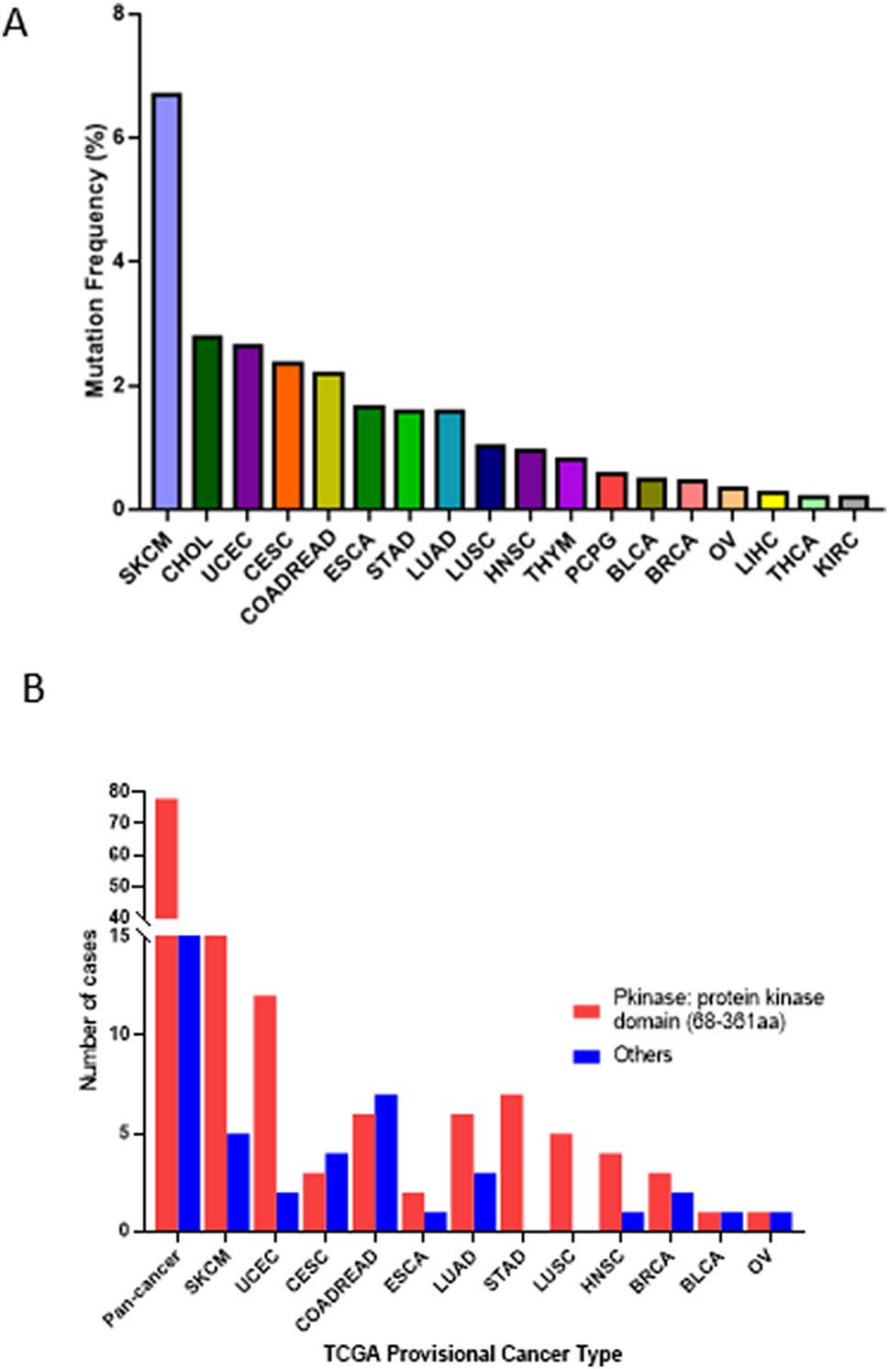
The mutation frequency and distribution of MEK1 in pancancer. (**A**) MEK1 mutation frequency in pancancer. (**B**) MEK1 mutation distribution belonging to different functional domains for all and the top 12 tumors.

Increasing evidence has demonstrated that mutation plays a promising role in regulation of gene expression and activation in mammalian cells^23,24^. Hence, we next wanted to evaluated the frequency and distribution of MEK1 mutation in pan-cancer. The 1.4% (108/7867) MEK1 total mutation frequency for patients was obtained from cBioPortral, and the range in the samples was from 36 (CHOL) to 1084 (BRCA) (Table 1). Across the 32 types of cancer, the SKCM mutation frequency was the highest (6.7%) and was more than twice as high as the second highest of CHOL (2.8%). The three highest frequencies were UCEC, CESC, and colorectal adenocarcinoma (COEADRE); and the frequencies were 2.6%, 2.4% and 2.2%, respectively. Conversely, TCTA and KIRC had low MEK1 mutation frequencies; and GBM, LGG, SARC, testicular germ cell tumor (TGCT), PRAD, PAAD and KIPR had almost no MEK1 mutation frequencies (Fig. 2A). These results may have some limitations in the sample and the range, but we believe the limitations may be solved perfectly because the importance attached to MEK1 and the accumulation of samples gradually increased.

**Table 1:** The MEK1 total mutation frequency obtained from cBiopotral.

	突变数	总数	Ratio (%)
LUAD	9	566	1.590106
LUSC	5	487	1.026694
STAD	7	440	1.590909
HNSC	5	523	0.956023
SKCM	30	448	6.696429
COADREAD	13	594	2.188552
THYM	1	123	0.813008
UCEC	14	529	2.646503
THCA	1	500	0.2
ESCA	3	182	1.648352
PCPG	1	178	0.561798
CHOL	1	36	2.777778
LIHC	1	372	0.268817
CESC	7	297	2.356902
OV	2	585	0.34188
KIRC	1	512	0.195313
BLCA	2	411	0.486618
BRCA	5	1084	0.461255

In the following work, we analyzed functional domains in which MEK1 mutations were located. The functional domains of MEK1 were classified as protein kinase domains (68–361 aa) and other domains using the Pfam database. The protein kinase domain is involved in cell metabolism though regulating cell signaling and activating other proteins by adding phosphate groups to protein substrates^19^. As Fig. 2B and Supplementary Table S1 show, in general, the mutations in the protein kinase domain (68–361 aa) were twice as many as others across the 32 types of cancer. The cancers whose mutations were most commonly distributed in the protein kinase domain were SKCM, UCEC, LUAD, STAD, LUSC, HNSC and BRCA. Importantly, STAD and LUAC mutations were all in the protein kinase domain. The opposite was that the mutations in CESC and COADREAD were distributed primarily in other domains whose functions were learned little, and their roles and profiles must continue to be explored.

Based on the classification of MEK1 functional mutation domains, our research was extended to further explore the defined mutation sites. Regarding the functional effects on protein coding, 108 MEK1 mutations were divided into three categories: missense mutations, truncating mutations and in-frame mutations. The most frequent mutation site, far more than the mutation of any other site, was located at 124 aa belonging to the protein kinase domain (Supplementary Figure S2A). Furthermore, the mutation of MEK1 in P124S/L almost exclusively occurred in SKCM (Supplementary Figure S2B). This finding implied that P124S/L was a mutational hotspot, having great potential to become a new therapeutic target for clinical treatment. As elaborated by Diamond et al., cobimetinib was significantly effective for the treatment of SKCM harboring the P124L mutation^25^. However, there was no FDA approval, and the mechanism of P124S/L mutation leading to tumors was thought to be unknown.

**Figure 3:**
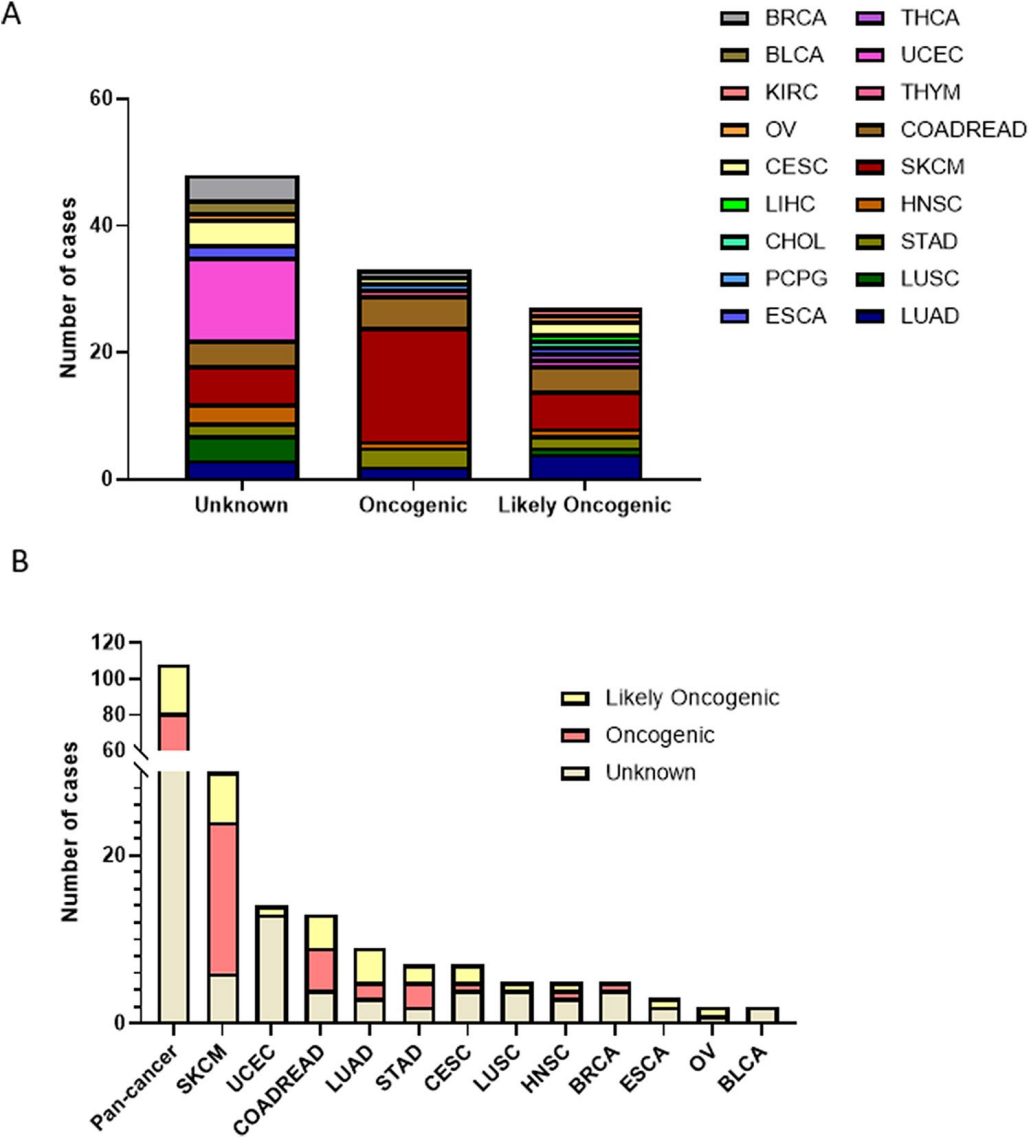
MEK1 mutation classification based on the functional impact. (**A**) MEK1 mutation classification based on the functional impact in pancancer. (**B**) MEK1 mutation classification based on the functional impact on all and top 12 tumors.

Next, we wanted to explore the biological significances of MEK1 mutations in tumorigenesis.108 MEK1 mutations were divided into three categories based on the oncogenic effects and predictive significance. They were unknown (48 mutations), oncogenic (33 mutations), and likely oncogenic (27 mutations). The majority of these mutations were unknown, indicating that it is a major challenge to determine their functions and that it is necessary for us to make greater efforts. Of note, the top four are SKCM (18/33), COADREAD (5/33), STAD (3/33) and LUAD (2/33) in oncogenic. Interestingly, in likely oncogenic, the top four are also SKCM (6/27), COADREAD (4/27), LUAD (4/27) and STAD (2/27) (Fig. 3A). Then, we analyzed the oncogenic effect separately according to the type of cancer. The oncogenic class accounted for the largest proportion in SKCM (18/30), COADREEAD (5/13) and STAD (3/7). The unknown class made up a large majority of UCEC (13/14), CESC (4/7), LUSC (4/5), HNSC (3/5), BRCA (4/5), ESCA (2/3) and BLCA (2/2) (Fig. 3B).

**Figure 4:**
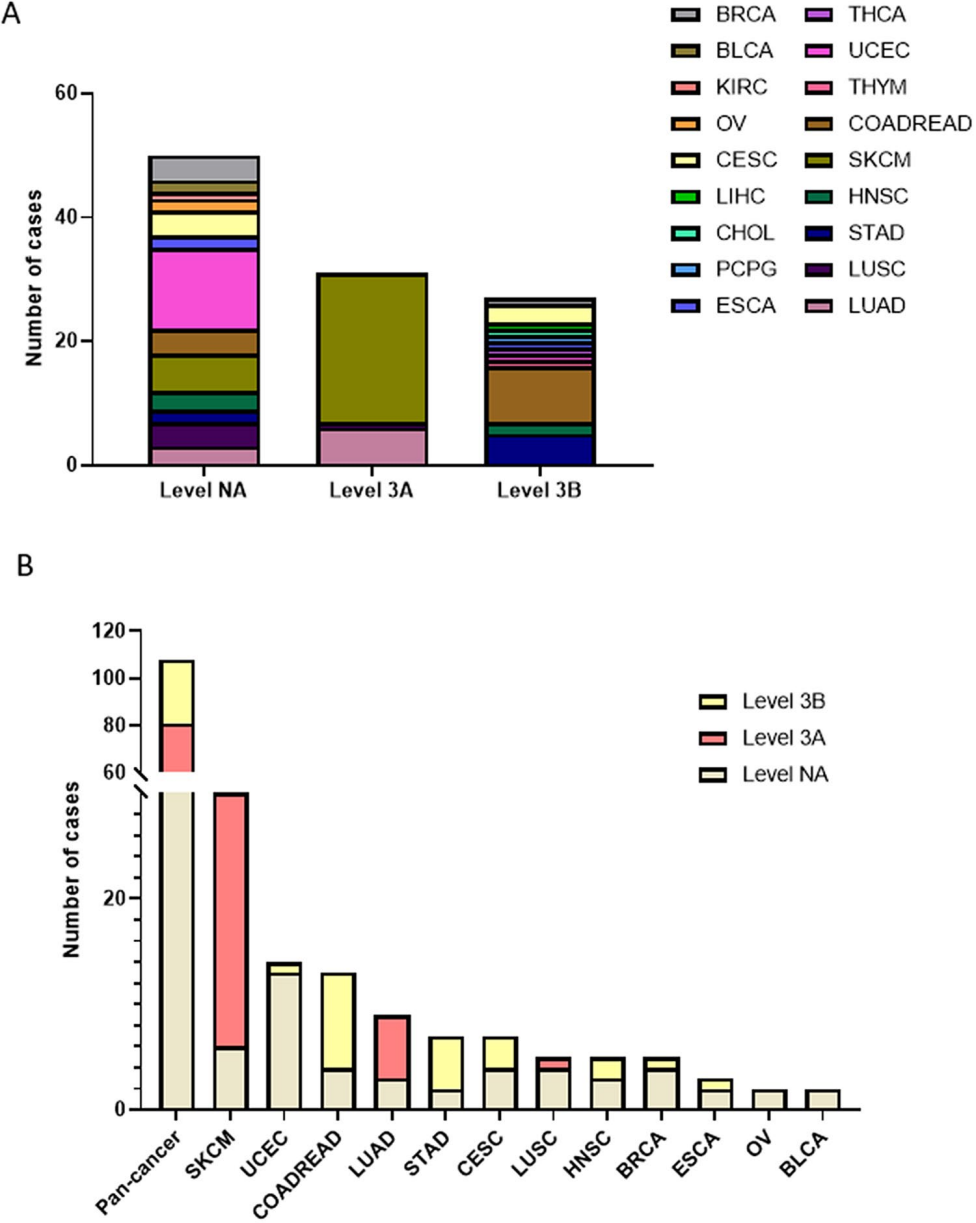
MEK1 mutation distribution by targeted therapy implication. (**A**) MEK1 mutation distribution by treatment level evidence as annotated in OncoKB. (**B**) MEK1 mutation distribution by targeted treatment in the top 12 types of cancer.

To identify the clinical targeted therapy significance of MEK1 mutations and practical clinical operability, we classified each MEK1 somatic mutation into three levels using mainly cBioPortal^17^ and OncoKB^26^. These levels were level NA (50 mutations), level 3A (31 mutations) and level 3B (27 mutations). Surprisingly, SKCM (24/31), LUAD (6/31) and LUAD (1/31) take up all of level 3A. The top four are COADREAD (9/27), STAD (5/27), CESC (3/27) and HNSC (2/27) in level 3B (Fig. 4A). Level 3A mutation represented the highest oncogenic evidence and was allowed to use off-label FDA-approved drugs or investigational agents not yet FDA approved for any indication^26^. Level NA mutations were primarily detected in ECEC (13/14), BRCA (5/6), OV (2/2) and BCLA (2/2) and had no targeted therapeutic significance. Level 3A mutations accounted for the largest proportion in SKCM (24/30) and AUAD (6/9). Level 3B mutations were the majority in COADROAD (9/13) and STAD (5/7) (Fig. 4B).

### MEK1 CNVs across 32 types of cancer

**Figure 5:**
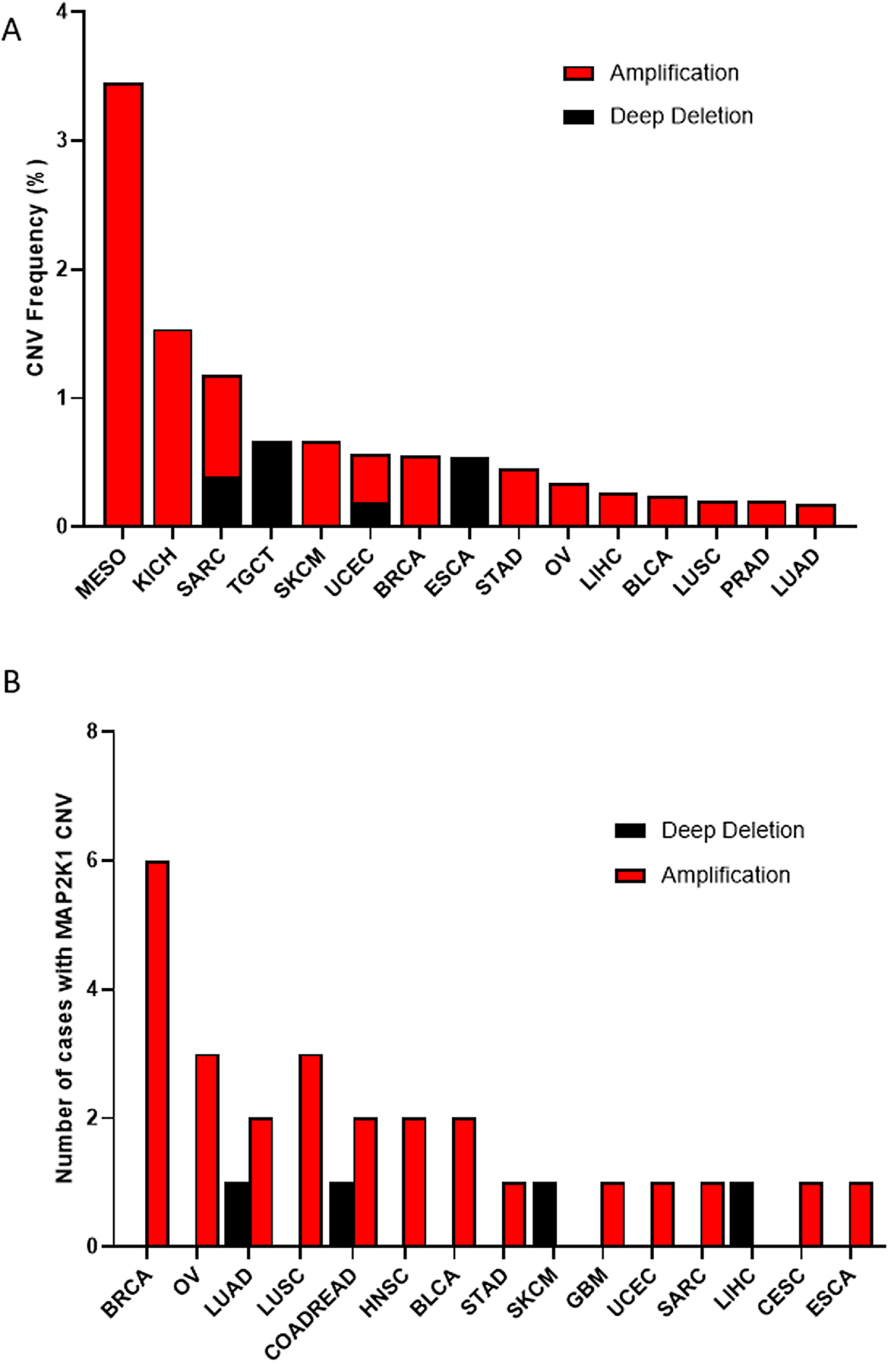
MEK1 copy number variant (CNV) distribution across 32 types of cancer. (**A**) MEK1 CNV frequency in 32 types of cancer. (**B**) MEK1 CNV distribution in top 15 types of cancer.

In addition to mutations, CNVs can also affect the expression and activation of cancer-associated genes, involving in the occurrence and development of many tumors^27,28^. MEK1 CNV frequency varied greatly in different tumors. There were almost amplification and deep deletion. The top three total MEK1 CVN frequencies were MESO (3.5%), KICH (1.5%) and SARC (1.2%). The bottom three were LUSC (0.2%), PRAD (0.2%) and LUAD (0.18%). Importantly, SARC and UCES had both amplification and deep deletion while only one type of CNV was observed in other cancers (Fig. 5A). From the point of view of the number of cases with MEK1 CNV, the numbers in descending order were BRCA, OV, LUAD, LUSC, COADREAD, etc. (Fig. 5B). To clarify the correlation between MEK1 CNVs and mRNA expression, we made a scatter diagram according to the data from cBioPortral. This result suggested that there was a positive correlation between MEK1 CNVs and mRNA expression across 32 types of cancer (r = 0.3768, p < 0.0001) (Supplementary Figure S3). Our work revealed that MEK1 CNVs play a crucial role in the mRNA expression and functions of tumors.

### Integrated MEK1 alterations (mutation and CNVs) in different tumors

**Figure 6:**
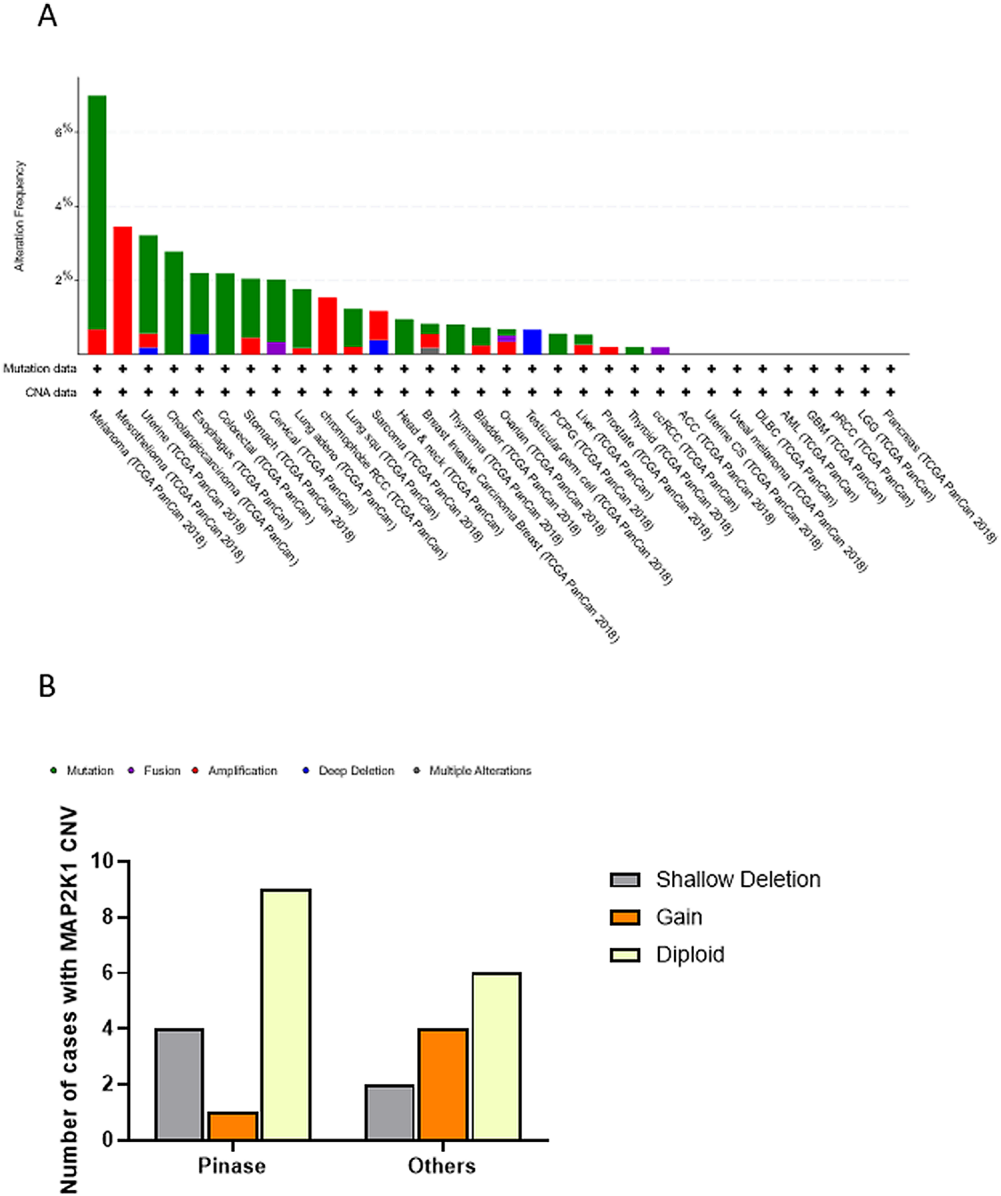
MEK1 alteration distribution in 32 types of cancer. (**A**) MEK1 alteration frequency in 32 types of cancer. (**B**) MEK1 CNV distribution located in different functional domains.

The total MEK1 alteration proportion accounted for approximately 7% across all types of cancer, and mutations were found more commonly in general. Nevertheless, the frequency varies markedly in different types of cancers. MEK1 alterations were more common in MESO, UCS, CHLO and ESCA. It is worth noting that there were exclusive amplifications in MESO, renal chromophobe cell carcinoma (RCCC) and prostate cancer (CRPC) while there were exclusive mutations in CHOL, COAD, HNSC and THCA. Furthermore, we observed neither MEK1 mutations nor MEK1 CNVs in ACC, UCS, UVM, DLBC, acute myeloid leukemia (AML), GBM, renal papillary cell carcinoma (PRCC), LGG and PAAD (Fig. 6A). Surprisingly, we observed that the MEK1 CNV frequency varied with the mutation sites. More than 50% of mutations in the protein kinase domain and other domains were accompanied by shallow MEK1 deletion and copy gain. The difference was that shallow deletion was dominant in the protein kinase domain while copy gain was dominant in other domains (Fig. 6B).

### MEK1 alterations and patient survival

**Figure 7:**
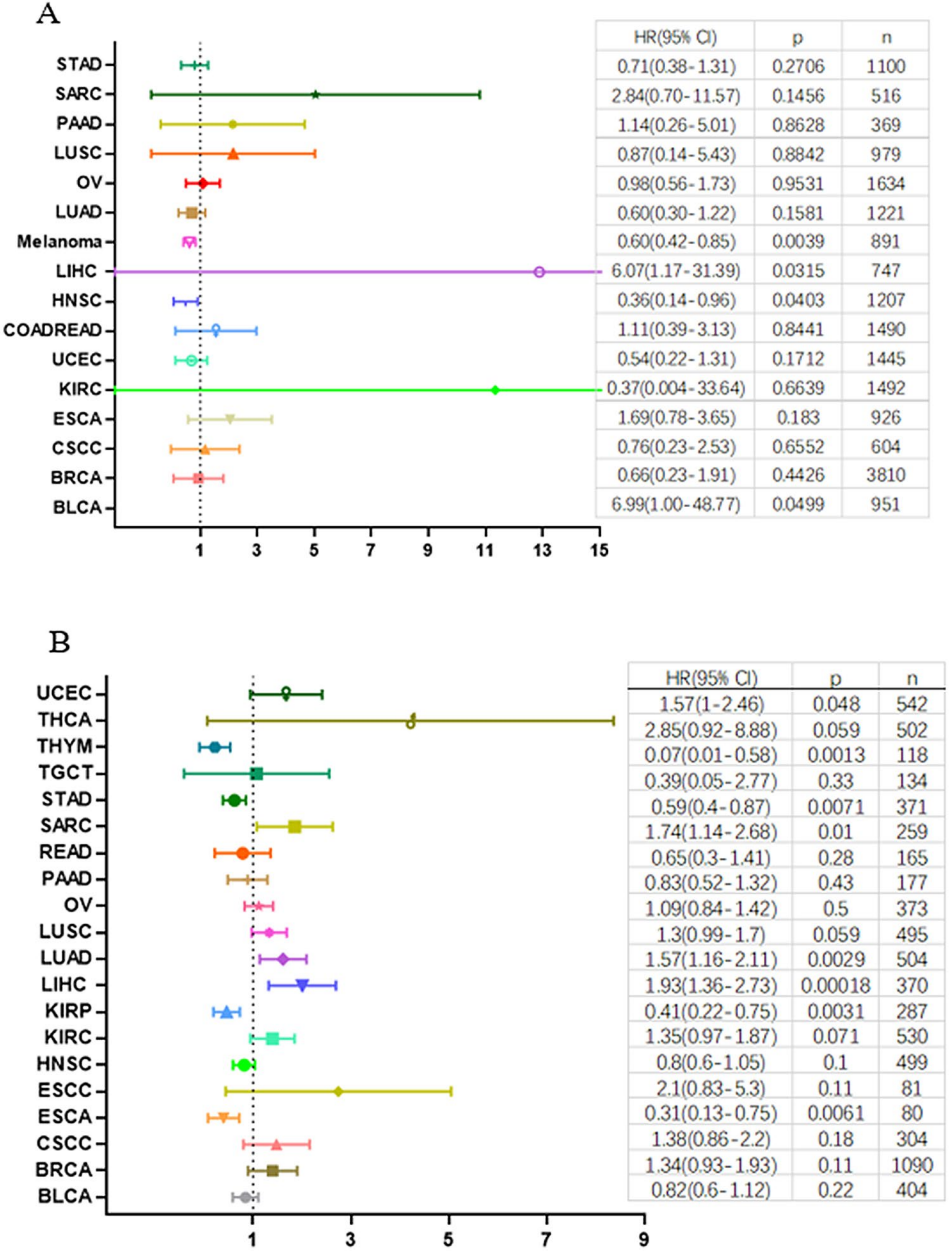
The relationship between MEK1 expression and patient overall survival (OS).

**Figure 8:**
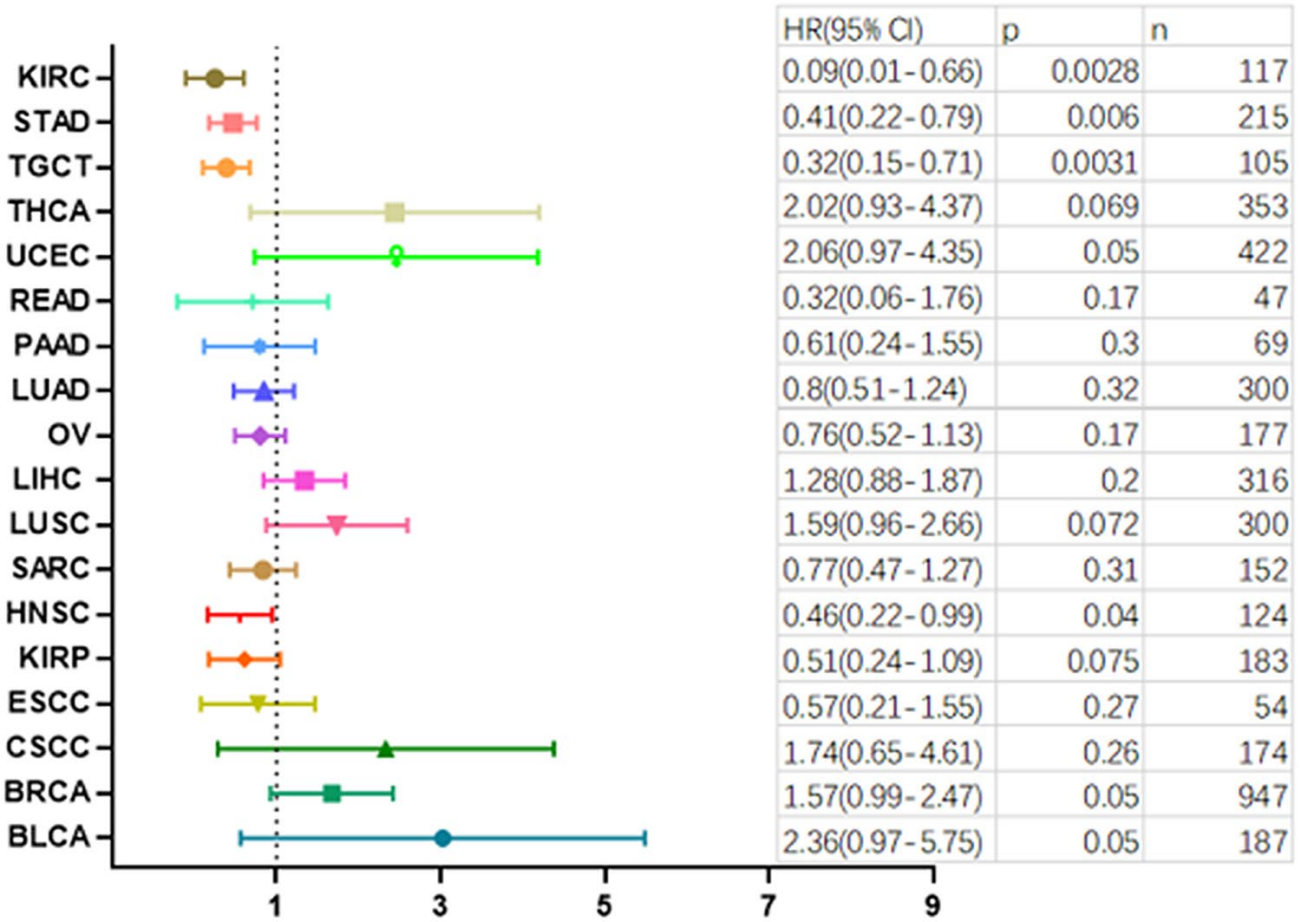
The relationship between MEK1 expression and patient progression-free survival (PFS).

The correlation between MEK1 mRNA expression and survival time was discussed to determine the clinical interpretation of MEK1 expression. According to the actual clinical needs, we analyzed patients with abnormal MEK1 expression overall time (OS) and progression-free survival (PFS) in individual tumors. Our results revealed that high MEK1 expression was a risk factor for patient OS in AUAD and LIHC. However, it was a protective factor for patient OS in melanoma, THYM, STAD, KIRP and ESCA (Fig. 7A,B). Next, the results of MEK1 expression and patient PFS are shown in Fig. 8A. High MEK1 expression was positively correlated with patient PFS in KIRC, STAD, TGCT and HNSC. Interestingly, a negative association between high expression and patient PFS was not observed.

## Discussion

In this research, we analyzed MEK1 in depth across 32 types of cancer from the following aspects: MEK1 expression, alterations, functional impact and clinical significance. Melanoma accounted for the largest proportion of alterations, and mutations were more common. Furthermore, MEK1 expression increased significantly, and mutations were mainly distributed in the protein kinase domain in SKCM. Overall, mutations occurred more often in the protein kinase domain, which was more likely to have therapeutic targets. Other types of cancer with high MEK1 alterations included UCEC, CHOL and COADREAD. There were several similarities such as the alteration frequency was approximately 2.8%, mutations were dominant and MEK1 expression decreased slightly compared with normal tissues. Notably, MESO had the second highest alterations in which amplification was considered to be a single type and the highest CNV frequency was observed but had no therapy targets. MEK1 expression in LUAD, KIRP, ESCA and LIHC decreased, which is associated with better prognosis. Conversely, the MEK1 expression in THYM, STAD, KIRC, TGCT and HNSC increased, which is associated with better prognosis. Regarding the other aspects of the expression profile, there were no alterations in ACC, UCS, UVM, DLBC, AML, GBM, PRCC, LGG or PAAD.

With deeper research and bioinformation database application development, a growing number of cancers have been found to be linked to MEK1. Previous studies have shown that high MEK1 expression is observed in various tumors. Moreover, many small molecule inhibitors targeting MEK1 have been developed. Malignant melanoma is an aggressive skin cancer^29^. Presently, malignant melanoma incidence is consistently growing, but mortality is not decreasing^30^. Many researchers have shown high MEK1 expression in BRAF-mutated melanoma due to the activation of RAF–MEK–ERK pathway. The MEK inhibitors, such as trametinib, cobimetinib and binimetinib, combined with the BRAF inhibitors has been approved for BRAF-mutated melanoma by FDA^14^. Long et al. revealed that dabrafenib plus trametinib was one of the established combination strategies for stage III BRAF-mutated melanoma patients. This clinical trial showed that 3-year overall and relapse-free survival rate in combination-therapy group were 86% and 58%, which are both significantly higher than that in placebo group^31^.

Patients with NF1 often have a high burden of genetic disorder owing to improved tumor predisposition syndrome and limited management options. This disease is a genetically heterogeneous disorder and leads to multiple manifestations and complications, such as pain, function damage, disfigurement and malignant transformation^32,33^. NF1 patients lack neurofibromin which leads to some dysregulated signaling molecules, including RAS, RAF, MEK1/2 and ERK^34^. Nowadays, MEK1 inhibitors can be used to treat the NF1 patients. Previous clinical researches indicated that treatment with selumetinib could obviously shrank the quantities of plexiform neurofibromas in patients with NF1. Moreover, selumetinib treatment could prevent the worsening of spinal canal distortion, reducing the need for surgical interventions and enhancing the clinical benefit^35^.

Histiocytic sarcoma is a rare, aggressive, and poorly understood hematopoietic neoplasm, and the majority of patients diagnosed live only 6 months^36^. One study found that histiocytic neoplasms might drive the RAF–MEK–ERK pathway and result in the overexpression of MEK1^37^. Naturally, trametinib was used to treat histiocytic neoplasms and preliminarily proved to be effective^38^. And a previous clinical research confirmed that the overall clinical benefit rate of patients with histiocytic neoplasms was significantly improved after trametinib treatment. More importantly, 72% patients (13/18) achieved a complete response, 17% patients (3/18) achieved a partial response and no patients had progressive disease^25^. Similarly, ARAF recurrent mutation excessively activating the RAF–MEK–ERK pathway leads to a central conducting lymphatic anomaly treatable with a MEK inhibitor^39^. MEK1 expression was increased in DLBC, and this result might be significantly related to driving the RAF–MEK–ERK pathway.

Emerging evidence that certain MEK1 mutations are thought to be carcinogenic has been presented in recent years. NCI-H1437 harboring the MEK1 Q56P mutation is a lung adenocarcinoma cell line. Preclinical research works have demonstrated that NCI-H1437 developed sensitivity to various MEK1 inhibitors. Similar situations were established in other cell lines. SNU-C1 belongs to the colorectal cancer cell line and harbors the MEK1 F53L mutation. OCUM-1 MEK1 harboring the MEK1 Q56P mutation is a gastric cancer cell line. These two cell lines were proven to be sensitive to MEK1 inhibitors^40^. Our findings showed that CORDREAD had high MEK1 expression, which was little associated with prognosis because these samples possibly had no MEK1 F53L mutation.

MEK1 is regulated by upstream factors and undergo a series of corresponding implications. Several seminal studies over the past decades have delineated upstream factors of MEK1 correlate with a greater risk of cancers, such as BRAF. It is well known that prooncogenic BRAF V600E-mutation could drive the malignant biological properties of melanoma cells^41^. Neurofibromin encoded by NF1 is a tumor suppressor and acts as a negative regulator of RAS activity. Lack of Neurofibromin causes RAS overactivation and RAS-driven cancers such as pancreatic ductal adenocarcinoma (PDA)^42,43^. These abnormal upstream signalings could eventually participate in tumorigenesis by regulating the cellular MEK1.

In this article, we discussed the expression, mutation frequency and CNVs of MEK1 in pan-cancers. However, the interrelationship between these three issues remains to be expected. Wee et al. gave a comprehensive analysis to identify the relationship between CNVs and gene expression in pan-cancers. This group reported that numerous oncogenes with frequent copy number of gain (CNG) were concentrated in the RAS-MAPK pathways^44^. The neuroendocrine carcinoma (NEC) patients harboring BRAF V600E mutations had 46.7% mutation frequency, which was significantly related to high levels of RAF, MEK and advanced tumor stages^29^.With the development of detection methods and scientific understanding, the findings in this study would represent a novel therapeutic strategy for patients with MEK1-related cancers.

## Conclusions

Overall, our study identified MEK1 characterizations across 32 TCGA types of cancer from four aspects including MEK1 expression, alterations, functional impact and clinical implications. Some alterations are closely related to tumorigenesis while others are strongly associated with targeted therapy. In addition, some kinds of cancer with high alteration frequency are involved in better prognosis, but others are just the opposite. Furthermore, some cancers with high expression or target sites can be treated with targeted drugs. Genomic profiling may provide significant insight into cancer treatment for using targeted drugs. Collectively, our results implicated the biological significances and applications of MEK1 in a variety of cancers. Future researches should be used to explore the potential regulation of MEK1 and evaluate the possible applications of MEK1 inhibitors in pan-cancer, which might provide promising therapeutic strategies and choices for cancer patients with MEK1 dysregulation.

## Supplementary Information

Supplementary Information.
