# Supplementary material for: Integrative pan-cancer analysis of MEK1 aberrations and the potential clinical implications
Source: Sci Rep. 2021 Sep 15;11:18366. doi: 10.1038/s41598-021-97840-0 (PMC8443600; doi:10.1038/s41598-021-97840-0)
Supplement: Supplementary file 1 — Supplementary Information. [file 41598_2021_97840_MOESM1_ESM.docx]

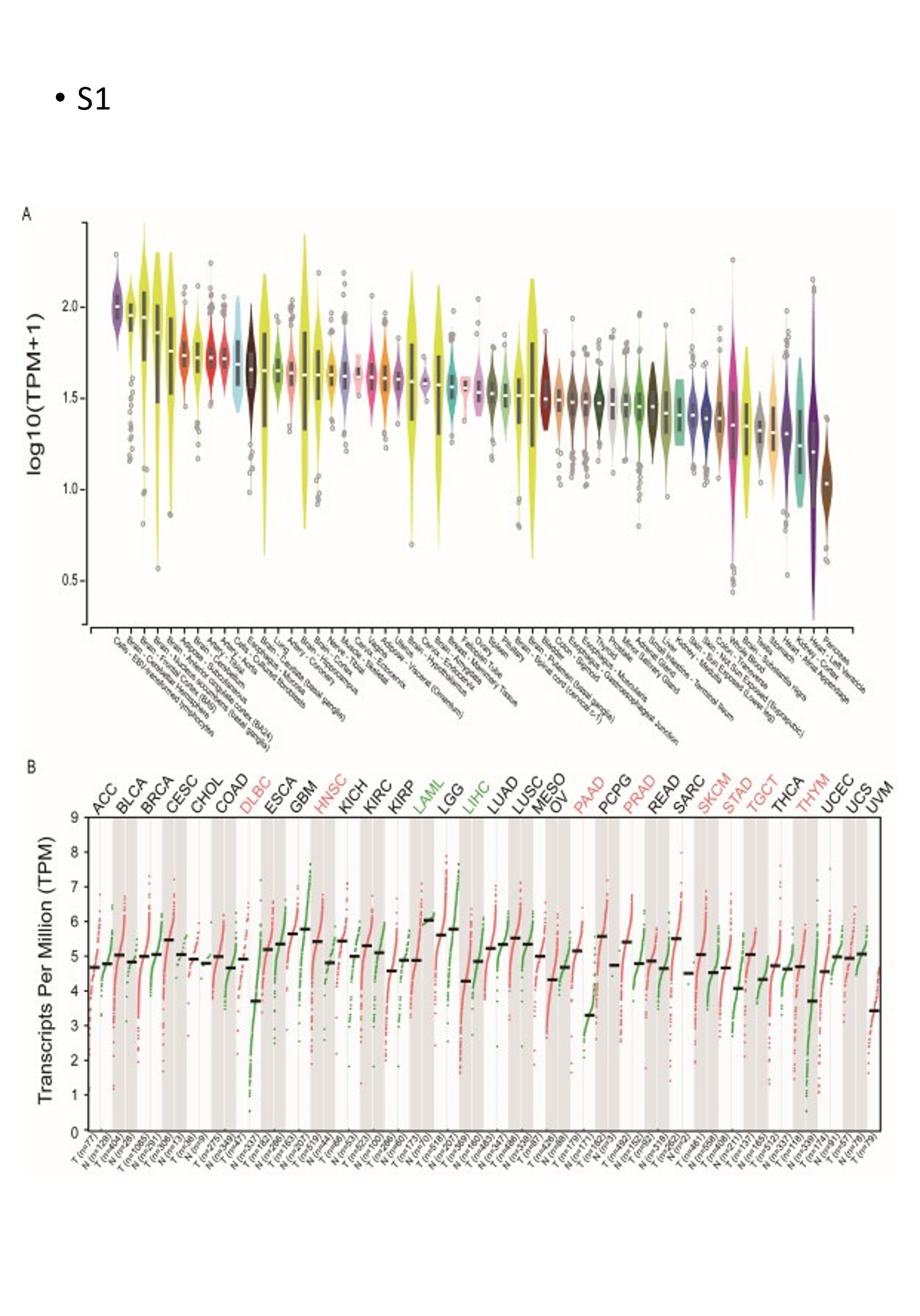


**Supplementary Figure S1.** MEK1 expression in different tissues. **(A)** MEK1 expression in normal tissues. **(B)** MEK1 expression between 32 types of tumor and paired normal tissues.


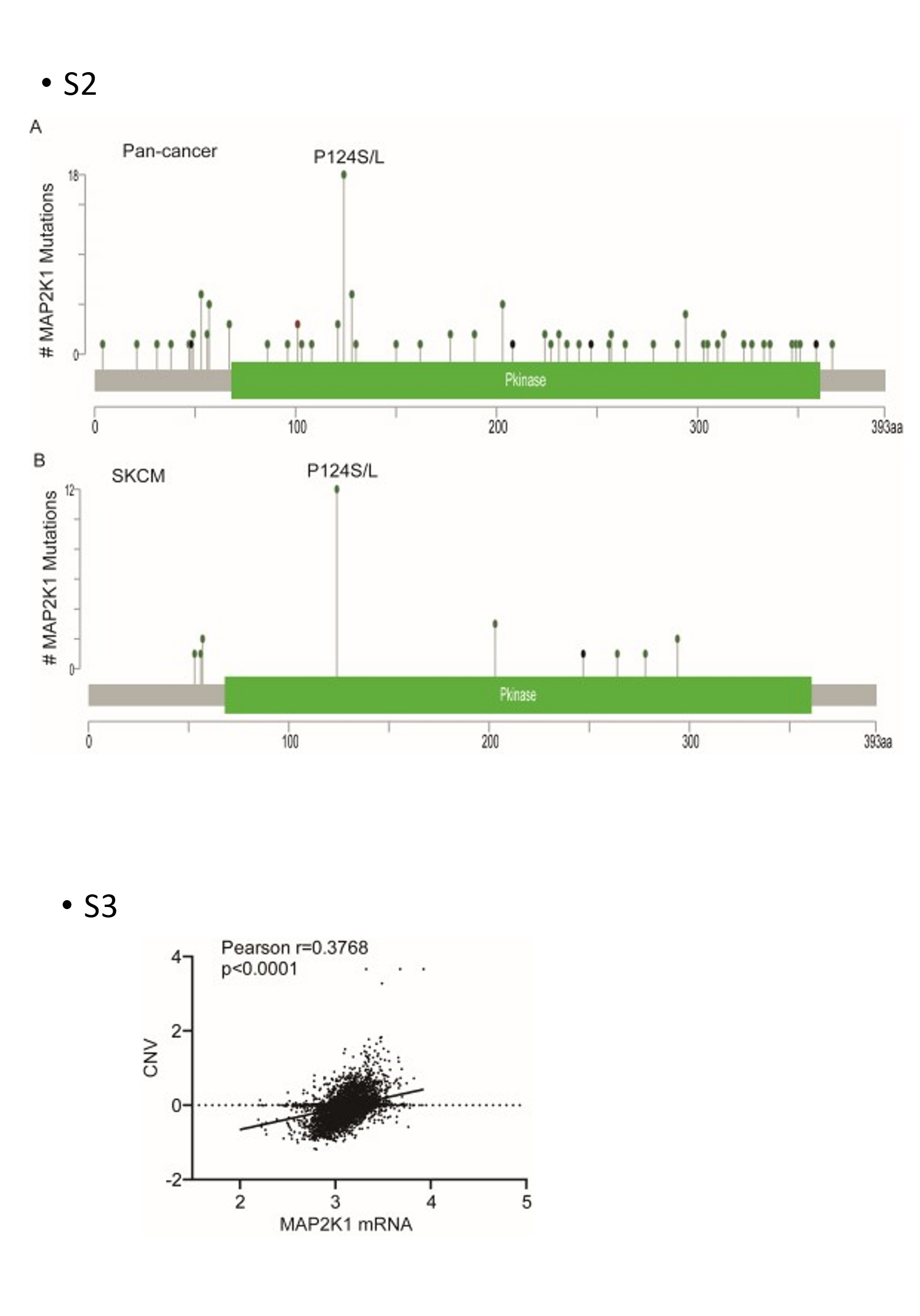


**Supplementary Figure S2.** MEK1 distribution of mutation sites. **(A)** MEK1 mutation site distribution in pancancer. **(B)** MEK1 mutation site distribution in skin cutaneous melanoma (SKCM).

**Supplementary Figure S3.** MEK1 mRNA expression was significantly correlated with MEK1 copy number variant (CNV).

**Supplementary Table 1.** Somatic mutation distribution in different functional domains

|  | Pkinase: protein kinase domain (68－361) | other | total |
| --- | --- | --- | --- |
| Pan-cancer | 78 | 30 | 108 |
| GBM |  |  |  |
| LGG |  |  |  |
| LUAD | 6 | 3 | 9 |
| LUSC | 5 |  | 5 |
| STAD | 7 |  | 7 |
| HNSC | 4 | 1 | 5 |
| SKCM | 25 | 5 | 30 |
| COADRE | 6 | 7 | 13 |
| THYM |  | 1 | 1 |
| UCEC | 12 | 2 | 14 |
| THCA | 1 |  | 1 |
| ESCA | 2 | 1 | 3 |
| PCPG |  | 1 | 1 |
| CHOL | 1 |  | 1 |
| LIHC | 1 |  |  |
| CESC | 3 | 4 | 71 |
| OV | 1 | 1 | 2 |
| KIRC |  | 1 | 1 |
| SARC |  |  |  |
| BLCA | 1 | 1 | 2 |
| TGCG |  |  |  |
| PRAD |  |  |  |
| PAAD |  |  |  |
| BRCA | 3 | 2 | 5 |
| KIRP |  |  |  |
